# Transmembrane domains of type III-secreted proteins affect bacterial-host interactions in enteropathogenic *E. coli*

**DOI:** 10.1080/21505594.2021.1898777

**Published:** 2021-03-17

**Authors:** Gershberg Jenia, Braverman Dor, Sal-Man Neta

**Affiliations:** The Shraga Segal Department of Microbiology, Immunology and Genetics, Faculty of Health Sciences, Ben-Gurion University of the Negev, Beer Sheva, Israel

**Keywords:** SctB, espb, scte, espd, epec, transmembrane domain, bacterial virulence, pore complex, type III secretion system

## Abstract

Many bacterial pathogens utilize a specialized secretion system, termed type III secretion system (T3SS), to translocate effector proteins into host cells and establish bacterial infection. The T3SS is anchored within the bacterial membranes and contains a long needle/filament that extends toward the host-cell and forms, at its distal end, a pore complex within the host membrane. The T3SS pore complex consists of two bacterial proteins, termed SctB and SctE, which have conflicting targeting indications; a signal sequence that targets to secretion to the extracellular environment via the T3SS, and transmembrane domains (TMDs) that target to membrane localization. In this study, we investigate whether the TMD sequences of SctB and SctE have special features that differentiate them from classical TMDs and allow them to escape bacterial membrane integration. For this purpose, we exchanged the SctB and SctE native TMDs for alternative hydrophobic sequences and found that the TMD sequences of SctB and SctE dictate membrane destination (bacterial versus host membrane). Moreover, we examined the role of the SctB TMD sequence in the activity of the full-length protein, post secretion, and found that the TMD does not serve only as a hydrophobic segment, but is also involved in the ability of the protein to translocate itself and other proteins into and across the host cell membrane.

## Introduction

The type III secretion system (T3SS) is a major bacterial virulence factor specialized in translocating effector proteins from the bacterial cytoplasm directly into the host cells. The T3SS consists of a cytoplasmic ring, a basal body that spans both inner and outer membranes, an external needle (and in some cases a long filament) that extends from the bacterial surface to bridge the extracellular space, and a pore complex within the host-cell membrane, named the translocon [1–6]. The translocon pore is formed by two hydrophobic proteins [7–9], named SctB and SctE, according to the unified Sct (secretion and cellular translocation) system [3,10]. These proteins are secreted by the T3SS together with the needle tip protein SctA (collectively called translocators), as an intermediate group following the secretion of the needle and inner rod proteins and preceding that of the effector proteins [3,11]. Once secreted to the extracellular environment, the SctA homo-oligomerizes to form a tip complex that caps the distal end of the needle, while SctB and SctE hetero-oligomerize and get embedded within the plasma membrane of the host cell, where they form a pore complex. This complex allows translocation of effectors directly into the host cytoplasm [12–14]. Therefore, single null mutants of *sctA, sctB* or *sctE* exhibit normal type III section (T3S), yet they are unable to infect host cells or cause disease in the animal models [15–20].

The SctB and SctE proteins are part of a unique group of secreted substrates, the transmembrane domain (TMD)-containing secreted proteins. These proteins are targeted for T3S to the extracellular environment, where they should remain soluble until they approach the host membrane and adopt a transmembrane orientation [21–23]. This ability to transfer from soluble to membrane-embedded is likely supported by their dual structural fluctuation from molten globule conformation in aqueous solution to transmembrane embedded ring-like structures with a stoichiometry of 6–8 subunits of each protein [8,22,24–27]. Before their secretion, the SctB and SctE proteins are associated with class II T3SS chaperones that prevent their premature folding and target them to the sorting platform of the T3S apparatus [28–33]. An ATPase found at the sorting platform associates with the chaperone-translocator complex to power chaperone release and the secretion of the translocators through the T3SS channel [34]. The chaperone-binding domains (CBD) of most SctB and StcE proteins are located within the 20–100 N-terminal residues of the proteins [35,36].

Per definition, TMD-containing secreted proteins have conflicting targeting indications. On the one hand, they contain an N-terminal secretion signal that guides them to extracellular secretion, and on the other hand they contain at least one TMD that can be recognized by the signal recognition particle (SRP) machinery, or less common membrane protein insertion machinery, to be delivered to the bacterial membrane [37–39]. The T3S signal is a non-cleavable sequence found within the first 20–50 amino acid residues of the protein sequence [40–43]. Although the T3S signals are of low sequence conservation, they are usually enriched in serine, threonine, isoleucine, and proline and are inherently unstructured [42,44,45]. It was recently reported that most TMD sequences of TMD-containing secreted proteins are of moderate hydrophobicity [46]. Insertion of such TMD sequences into reporter systems revealed that these TMDs have a reduced propensity to be targeted and integrated into the bacterial membrane [46].

In this study, we utilized the full-length SctB and SctE proteins of enteropathogenic *E. coli* (EPEC), the causative agent of pediatric diarrhea [47], to investigate whether their TMD sequences enable them to escape integration into the bacterial membrane and to determine whether these TMDs are involved in the activity of the protein post-secretion. The proteins, named EspB (SctB) and EspD (SctE), are encoded within the pathogenicity island of EPEC, termed the locus of enterocyte effacement (LEE). The LEE contains seven major operons (designated *LEE1*-7); *espB* and *espD* are encoded in *LEE4*, together with the *espA* translocator (*sctA*), which forms the long extracellular filament at the end of the T3SS needle [48–51]. In this study, we found that the TMDs of EspB and EspD encode critical information regarding their final membrane destination (bacterial or plasma membrane) and that the TMD of EspB is involved in post-secretion events that are crucial for protein activity within the host-cell membrane.

## Results

**Figure 1.**
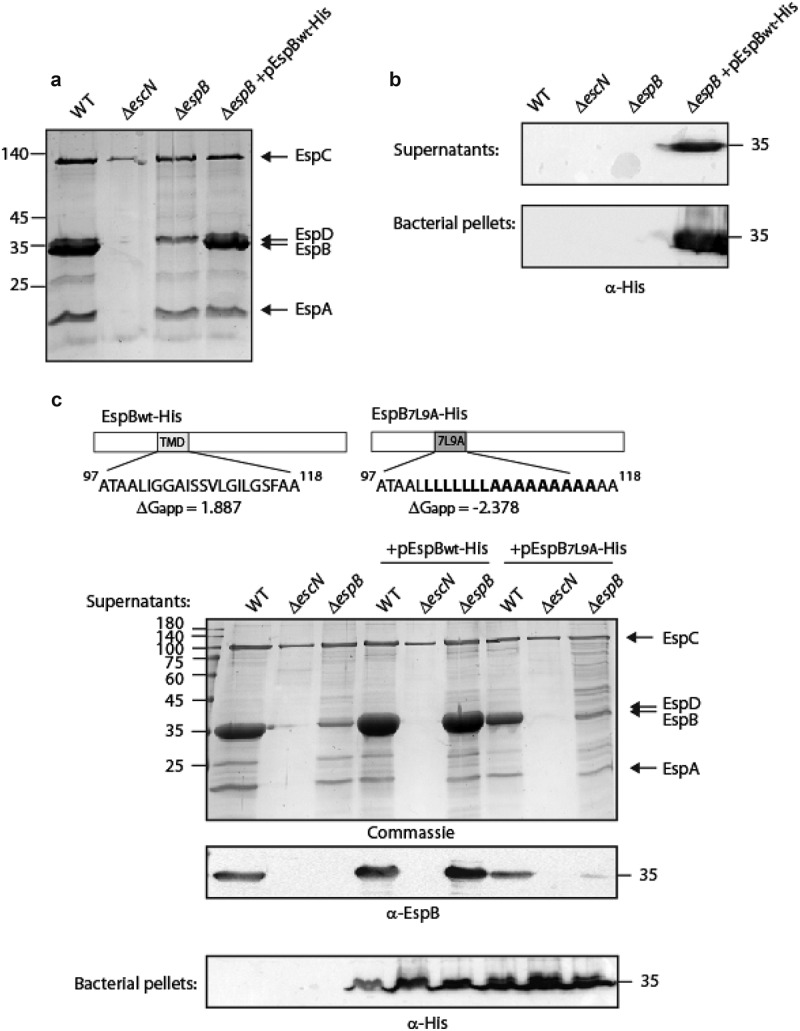
**The TMD sequence of EspB is critical for the ability of the protein to be secreted by the T3SS**. (a) Protein secretion profiles of EPEC strains grown under T3SS-inducing conditions: wild-type (WT) EPEC, Δ*escN* (a T3SS ATPase mutant), Δ*espB*, and Δ*espB* expressing EspB_wt_-His. The secreted fractions were concentrated from the supernatants of bacterial cultures and analyzed by SDS-PAGE and Coomassie blue staining. The T3SS-secreted translocators EspA, EspB, and EspD are marked on the right of the gel. Also indicated is the location of EspC, which is not secreted via the T3SS. (b) Bacterial pellets and supernatants were analyzed by SDS-PAGE and western blot with an anti-His antibody to confirm EspB_wt_-His expression and secretion. (c) Schemes of the EspB_wt_ and EspB_7L9A_ proteins with their corresponding TMD sequences and the calculated apparent free energy differences(ΔG_app_). Protein secretion profiles and bacterial pellets of EPEC WT, Δ*escN*, and Δ*espB* strains only or expressing either EspB_wt_-His or EspB_7L9A_-His, grown under T3SS-inducing conditions were analyzed similar to panel A

**Alteration of EspB (SctB) TMD sequence reduces protein secretion**. To characterize the EspB protein, we labeled it with a C-terminal His-tag and examined the protein ability to be secreted through the T3SS, similarly to the native protein. T3S in EPEC is characterized by the ability of the bacteria, grown under T3SS-inducing conditions, to secrete three T3SS translocators (EspA, EspB, and EspD) into the culture supernatant. Indeed, we observed that WT EPEC demonstrated T3SS activity while the Δ*escN* mutant strain, with a deletion of the T3SS ATPase gene, did not secrete translocators (Figure 1a). As expected, the Δ*espB* strain showed lower protein intensity at the size of EspB (~33 kDa), and significant EspB_wt_-His secretion was observed for the Δ*espB* strain overexpressing the labeled protein (Figure 1a). Since the EspB and EspD proteins are of similar size (33 and 39 kDa, respectively), they are difficult to separate by SDS-PAGE and Coomassie staining. Therefore, we analyzed the bacterial pellets and supernatants by western blot using an anti-His antibody. EspB_wt_-His expression and secretion were detected only in the Δ*espB* strain carrying the pEspB_wt_-His vector (Figure 1b). These results confirmed that labeling EspB at its C-terminus does not interfere with the protein secretion through the T3SS.

To determine whether the TMD of EspB contains features that differentiate it from classical TMDs and allow the EspB protein to be targeted for secretion instead of membrane localization, we replaced the original TMD of EspB by an alternative hydrophobic sequence. For this purpose, we exchanged the 16 core residues of EspB TMD by a hydrophobic sequence of seven leucines followed by nine alanines (7L9A) (Figure 1c). The 7L9A sequence, embedded between 5 hydrophobic amino acids of the original TMD, was previously shown to be sufficiently hydrophobic to support protein integration into the bacterial membrane [52–55]. The WT and TMD-exchanged constructs (EspB_wt_-His and EspB_7L9A_-His) were transformed into EPEC WT, Δ*escN*, and Δ*espB* EPEC strains, grown under T3SS-inducing conditions and examined for their ability to secrete EspB. While EspB_wt_-His expressed in the Δ*espB* mutant was secreted to a similar level as the native EspB of EPEC WT, the secretion of EspB_7L9A_-His expressed in the Δ*espB* strain was significantly reduced (Figure 1c). Since EspB_wt_-His and EspB_7L9A_-His showed a similar expression level in all EPEC strains (in bacterial pellets), we excluded the possibility that the reduced secretion of EspB_7L9A_-His was due to lower protein expression. As expected, no secretion of EspB (native, EspB_wt_-His, or EspB_7L9A_-His) was observed in any of the Δ*escN* strains. Interestingly, western blot analysis of the supernatants of EPEC WT revealed that expression of EspB_7L9A_-His in the WT EPEC strain partially inhibited EspB secretion (Figure 1c).

**Figure 2.**
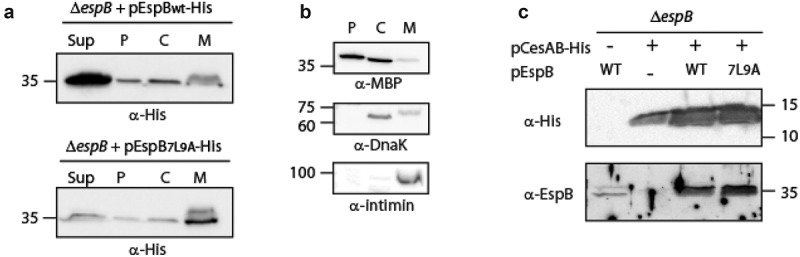
**The localization of EspB and its interaction with CesAB**. (a) Δ*espB* EPEC expressing either EspB_wt_-His or EspB_7L9A_-His were grown under T3S-inducing conditions, supernatants were collected, and whole-cell extracts were fractionated into periplasmic (p), cytoplasmic (c), and membranal (m) fractions. The samples (30 µg) were analyzed by SDS-PAGE and western blotting using an anti-His antibody. (b) To confirm correct bacterial fractionation, the western blots were probed with anti-DnaK (cytoplasmic marker), anti-MBP (periplasmic marker), and anti-intimin (membranal marker) antibodies. (c) Elution fractions of EPEC Δ*espB* expressing EspB_wt_ alone, CesAB-His alone or CesAB-His in combination with EspB_wt_ or EspB_7L9A_, were loaded on SDS-PAGE and analyzed by western blotting with anti-His and anti-EspB antibodies

**The TMD sequence influences EspB localization**. To determine if the exchange of the original EspB TMD with the 7L9A sequence affected the localization of the protein, EPEC Δ*espB* that over-express either EspB_wt_-His or EspB_7L9A_-His were grown under T3SS-inducing conditions. Supernatants were collected, and whole-cell extracts were fractionated into cytoplasmic, periplasmic, and membranal fractions. Western blot analysis with an anti-His antibody revealed that while EspB_wt_-His was found mostly in the supernatant fraction, EspB_7L9A_-His was enriched in the membrane fraction and was reduced in the supernatant compared to EspB_wt_-His (Figure 2a). Correct bacterial fractionation was confirmed by western blots probed with anti-DnaK (a cytoplasmic marker), anti-MBP (a periplasmic marker), and anti-Intimin (a membranal marker) antibodies (Figure 2b). Overall, the fractionation results suggest that the TMD sequence of EspB regulates protein localization by promoting protein secretion rather than membrane integration.

Previous reports showed that several class I T3SS chaperones mask hydrophobic regions of effectors to prevent their intra-bacterial aggregation or improper membrane localization [46,56,57]. To examine whether the replacement of the EspB TMD sequence by the 7L9A sequence disrupts the interaction of the protein with its chaperone, CesAB, we expressed unlabeled EspB_wt_ and EspB_7L9A_ in the presence of CesAB-His. The bacterial strains were grown under non-T3SS inducing conditions to prevent secretion of EspB to the extracellular environment. Whole cell lysates were incubated with Ni-NTA resin to pull down CesAB-His along with its binding partners. SDS-PAGE and western blot analysis of the eluted samples, using anti-His and anti-EspB antibodies, demonstrated that both EspB_wt_ and EspB_7L9A_ co-eluted with CesAB-His to a similar level (Figure 2c). These results suggested that the secretion deficiency of EspB_7L9A_ was not due to a reduced interaction with the CesAB chaperone. Although a few translocator chaperones were previously shown to bind the TMDs of their cognate substrates in addition to their CBD [46,56–58], we found that the TMD sequence of EspB is not critical for EspB-CesAB interaction.

**Figure 3.**
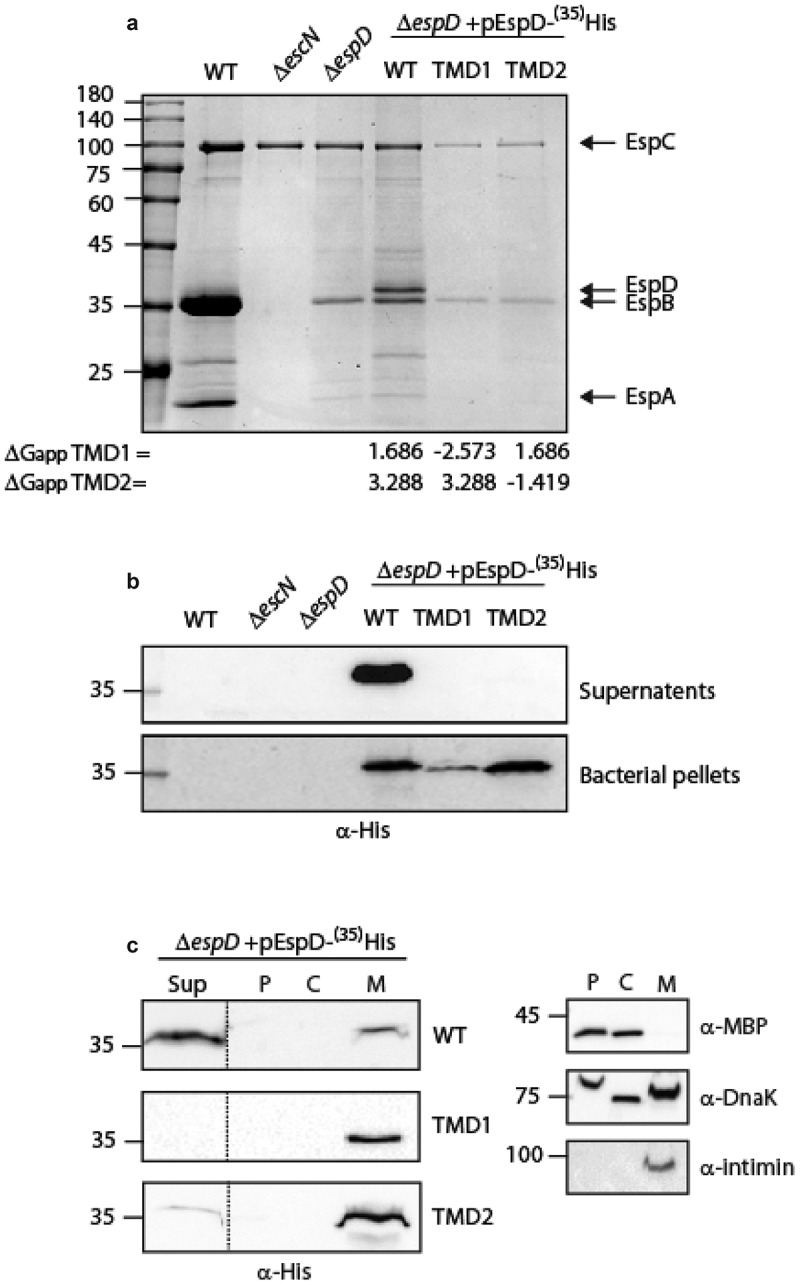
**The TMD sequences of EspD are crucial for the secretion of the protein by the T3SS**. (a) Protein secretion profiles of EPEC strains grown under T3SS-inducing conditions: WT EPEC, Δ*escN*, Δ*espD*, and Δ*espD* expressing either EspD_wt_-^35^His, EspDTMD1-^35^His, and EspDTMD2-^35^His. The secreted fractions were concentrated from the supernatants of bacterial cultures and analyzed by SDS-PAGE and Coomassie blue staining. The T3SS-secreted translocators EspA, EspB, and EspD are marked on the right of the gel. Also indicated is the location of EspC, which is not secreted via the T3SS. The calculated ΔG_app_ of each TMD sequence is presented. (b) Bacterial pellets and supernatants were analyzed by SDS-PAGE and western blot with an anti-His antibody to confirm EspD expression and secretion. (c) Δ*espD* EPEC expressing either EspD_wt_-^35^His (labeled WT), EspD_TMD1_-^35^His (labeled TMD1), or EspD_TMD2_-^35^His (labeled TMD2) were grown under T3S-inducing conditions, supernatants were collected, and whole-cell extracts were fractionated into periplasmic (p), cytoplasmic (C), and membranal (m) fractions. The samples (30 µg) were analyzed by SDS-PAGE and western blot using an anti-His antibody. To confirm correct bacterial fractionation, the western blots were probed with anti-DnaK (cytoplasmic marker), anti-MBP (periplasmic marker), and anti-intimin (membranal marker) antibodies

**Exchange of EspD (SctE) TMD sequences alters EspD secretion**. To determine whether the involvement of the EspB TMD in the secretion of the protein is unique to EspB, or represents a broader phenomenon, we examined the involvement of the EspD TMDs in its secretion. For this purpose, we cloned EspD_wt_-^35^His, EspDTMD1-^35^His, or EspDTMD2-^35^His which contain a replacement of the 16 core residues of TMD1 or TMD2 of EspD by the 7L9A sequence, respectively. The vectors encoding EspD_wt_-^35^His, EspDTMD1-^35^His, or EspDTMD2-^35^His were then transformed into Δ*espD*, and the strains were examined for their ability to secrete the EspD protein (WT or TMD-exchanged). Expression of EspD_wt_-^35^His within the Δ*espD* strain restored secretion of EspD but showed significant reduction in EspB secretion (Figure 3a). The effect of EspD expression on EspB secretion was previously reported and suggested to be associated with sensitivity of EspB secretion to *espD* gene copy number [18]. While EspD_wt_-^35^His was detected in the supernatant sample of Δ*espD* carrying pEspD_wt_-^35^His, no secretion of either EspD_TMD1_-^35^His or EspD_TMD2_-^35^His was observed (Figures 3a and 3b). Examination of EspD expression within the bacterial pellet revealed that both EspD WT and TMD-exchanged versions are expressed, thus excluding the possibility that the inability to be secreted is due to poor protein expression (Figure 3b). Moreover, examination of the localization of the EspD WT and TMD-exchanged versions revealed that while EspD_wt_-^35^His was mostly found in the supernatant, EspD_TMD1_-^35^His and EspD_TMD2_-^35^His localized mainly to the bacterial membrane (Figure 3c). These results suggested that similarly to EspB, the TMDs of EspD contain special features that allow the protein to avoid being integrated into the bacterial membrane and get secreted instead.

**Figure 4.**
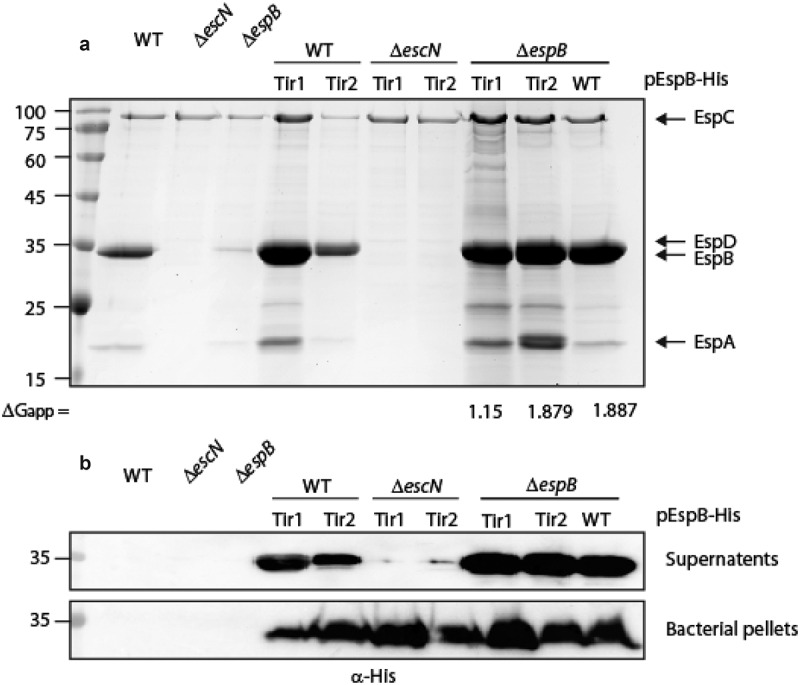
**Replacement of EspB TMD by TMD sequences of Tir preserves the ability of EspB to be secreted by the T3SS**. (a) Protein secretion profiles of EPEC WT, Δ*escN*, and Δ*espB* strains, and the same strains expressing either EspB_wt_-His, EspB_Tir1_-His, or EspB_Tir2_-His grown under T3SS-inducing conditions. The secreted fractions were concentrated and analyzed by SDS-PAGE and Coomassie blue staining. The location of the T3SS translocators EspA, EspB, EspD, and EspC (which is not secreted via the T3SS), are marked on the right of the gel. The calculated ΔG_app_ of each TMD sequence is presented. (b) Bacterial pellets and supernatants were analyzed by SDS-PAGE and western blot with an anti-His antibody. EspB_Tir1_-His and EspB_Tir2_-His were detected in the supernatants of WT and Δ*espB* strains, but not when expressed in the Δ*escN* mutant

**The TMDs of type III-secreted transmembrane proteins are interchangeable for secretion purposes**. To determine if the TMD sequences of TMD-containing secreted-proteins have common features that enable them to escape the co-translational targeting pathway, we replaced the EspB TMD sequence by the TMDs of another TMD-containing secreted protein, termed Tir, which is the first and the most abundant effector to be translocated to host cells [59–61]. Tir contains two TMDs and adopts a hairpin topology, with both its N- and C-termini positioned within the host cytoplasm [62]. The calculated apparent free energy differences (ΔG_app_) for TMD insertion of Tir1 and Tir2 TMDs into the membrane are 1.15 and 1.879, respectively, which is closer to the value of EspB TMD (1.887) compared to the value of 7L9A TMD sequence (−2.378) [63]. We replaced the TMD of EspB with each of the Tir TMDs and examined the ability of these TMD-exchanged proteins to be secreted by the T3SS. In contrast to EspB_7L9A_-His, the levels of secreted EspB_Tir1_-His and EspB_Tir2_-His were similar to that of EspB_wt_-His when expressed in the Δ*espB* strain (Figures 4a and 4b). As expected, no secretion was observed for these EspB TMD-exchanged versions when they were expressed in the Δ*escN* mutant strain, confirming that their secretion was T3SS-dependent (Figures 4a and 4b).

**Figure 5.**
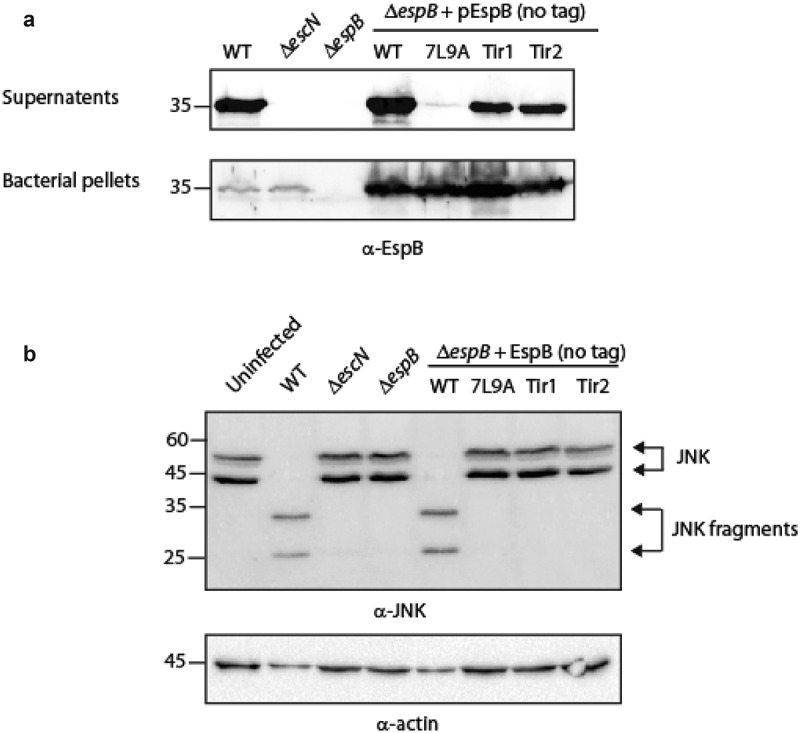
**The TMD sequence of EspB is crucial for the activity of the protein**. (a) Bacterial pellets and supernatants of EPEC WT, Δ*escN*, Δ*espB*, and Δ*espB* that express unlabeled EspB_wt_, EspB_7L9A_ EspB_Tir1_, or EspB_Tir2_ were grown under T3SS-inducing conditions. The samples were analyzed by SDS-PAGE and western blot with an anti-EspB antibody. (b) Proteins extracted from HeLa cells infected with WT, Δ*escN*, Δ*espB*, and Δ*espB* expressing EspB_wt_, EspB_7L9A_ EspB_Tir1_ or EspB_Tir2_. The samples were subjected to western blot analysis using anti-JNK and anti-actin (loading control) antibodies. JNK and its degradation fragments are indicated at the right of the gel. WT EPEC showed massive degradation of JNK relative to the uninfected sample and the samples infected with Δ*escN* or Δ*espB* mutant strains. The EPEC Δ*espB* strain complemented with EspB_wt_ showed a similar JNK degradation profile as WT EPEC, while the Δ*espB* strain complemented with EspB_7L9A_ EspB_Tir1_ or EspB_Tir2_ showed JNK degradation profiles similar to those of Δ*escN* and Δ*espB* strains

**EspB TMD is crucial for the activity of the T3SS complex post secretion**. We aimed to determine if the sequence of EspB TMD is crucial for the protein function post-secretion or if it is needed only for its hydrophobic nature, to support transmembrane orientation. To this end, we examined the ability of EspB TMD-exchanged versions to complement the deficiency of the Δ*espB* mutant strain to translocate effectors into host cells. We first examined whether a plasmid-expressed EspB_wt_-His can complement the Δ*espB* phenotype. For this purpose, we infected HeLa cells with various EPEC strains (WT, Δ*escN*, Δ*espB*, and Δ*espB* expressing EspB_wt_-His) and examined the cleavage pattern of c-Jun N-terminal kinase (JNK), a host protein that is cleaved by a translocated EPEC effector, called NleD [64]. WT EPEC induced extensive degradation of JNK, relative to the uninfected sample and the samples infected with the Δ*escN* or Δ*espB* mutant strains (Fig. S1). EPEC Δ*espB* that express EspB_wt_-His showed no JNK degradation, thus indicating that the labeled EspB_wt_-His protein, although secreted by the T3SS, is unable to functionally complement Δ*espB* translocation activity (Fig. S1). To determine whether the C-terminal labeling of EspB resulted in the inability of the protein to complement Δ*espB* translocation, we cloned a plasmid-expressed unlabeled EspB (pEspB_wt_). The plasmid was transformed into the Δ*espB* strain, and the strain was examined for its ability to translocate NleD into HeLa cells. We found that unlabeled EspB was able to complement the Δ*espB* phenotype and translocate NleD into HeLa cells (Fig. S1). Next, we cloned the unlabeled TMD-exchanged versions (EspB_7L9A_, EspB_Tir1_ and EspB_Tir2_) and transformed the plasmids into Δ*espB*. Examination of EspB_Tir1_ and EspB_Tir2_ secretion revealed that these TMD-exchanged versions have a similar secretion level as EspB_wt_ that is expressed from the same plasmid while the EspB_7L9A_ showed negligible secretion (Figure 5a).

Following the observation of proper EspB secretion, we examined the ability of Δ*espB* strains expressing either EspB_7L9A_, EspB_Tir1_ or EspB_Tir2_ to translocate NleD into host cells. We concluded that all three strains were unable to translocate the NleD effector into HeLa cells since they presented a similar JNK pattern as the uninfected sample and the samples of HeLa cells infected with Δ*escN* and Δ*espB* strains (Figure 5b). These results suggested that the TMD sequence of EspB is involved not only in the ability of the protein to be secreted but also in the activity of the protein post secretion.

**Figure 6.**
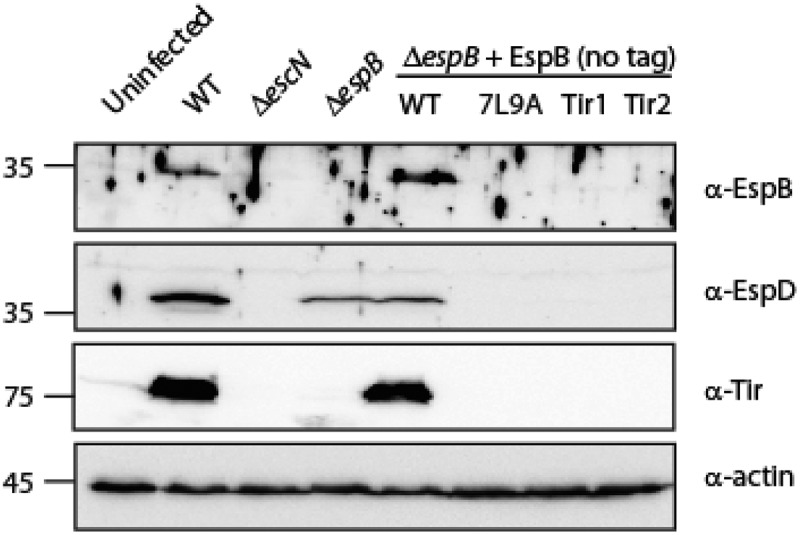
**EspB’s TMD is critical for the ability of EspB, EspD and Tir to translocate into host cell membranes**. HeLa cells were infected with EPEC WT, Δ*escN*, Δ*espB*, and Δ*espB* that express EspB_wt_, EspB_7L9A_, EspB_Tir1_, or EspB_Tir2_, washed, and lysed. Cellular samples were subjected to SDS-PAGE and western blot analysis using anti-EspB, anti-EspD, anti-Tir, and anti-actin (loading control) antibodies. EspB, EspD and Tir translocation was detected in HeLa cells infected with EPEC WT or Δ*espB* expressing EspB_wt_, but not in HeLa cells infected with Δ*espB* expressing either EspB_7L9A_, EspB_Tir1_ or EspB_Tir2._

**EspB TMD is involved in the ability of EspB, EspD and Tir to translocate into host membranes**. To examine whether Δ*espB* strains expressing either EspB_Tir1_ or EspB_Tir2_ were defective not only in their ability to translocate NleD but also in their ability to translocate other effectors and translocators, we collected host-cells following bacterial infection, lysed them, and tested them for EspB, EspD, and Tir presence. While translocated EspB was observed in cells infected by WT EPEC and Δ*espB* strain that expresses EspB_wt_ protein, no translocation was observed in cells infected with Δ*espB* that express either EspB_7L9A_, EspB_Tir1_ or EspB_Tir2_ (Figure 6). These results suggested that the TMD of EspB is crucial for the ability of the protein to insert properly into the plasma membrane and facilitate effector translocations.

Translocation of EspD was observed in cells infected by WT EPEC and to a lesser extent in cells infected by Δ*espB* strain (Figure 6). This result concurs with a previous study that reported that the major translocator (SctE) translocates into host cells independently of the minor translocator (SctB) [65]. Similarly, reduced EspD translocation was also observed in cells infected with the Δ*espB* strain that expressed EspB_wt_ (Figure 6). Interestingly, infection of cells with Δ*espB* strain expressing either EspB_7L9A_, EspB_Tir1_ or EspB_Tir2_ resulted in negligible translocation of EspD to the host cells, thus implying that the presence of non-translocated EspB can interrupt with the ability of EspD to insert into the host membrane.

Tir translocation was observed in cells infected by WT EPEC and Δ*espB* strain that expressed EspB_wt_, while no translocation was observed for the Δ*espB* strain that expressed either EspB_7L9A_, EspB_Tir1_ or EspB_Tir2_ (Figure 6). Overall, our results demonstrated that alteration of the TMD of EspB impairs the translocation of EspB and sequentially that of EspD and Tir.

**Figure 7.**
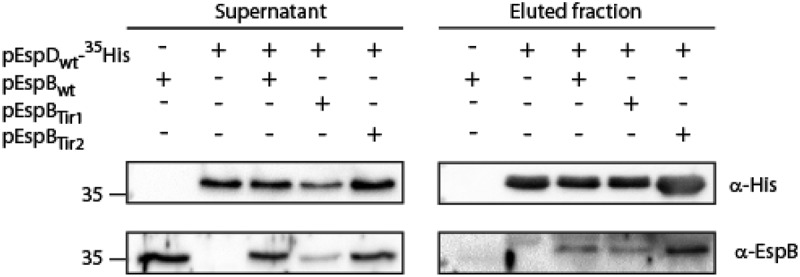
**EspB TMD is not involved in EspB-EspD interaction**. Supernatants of EPEC Δ*espB* expressing EspB_wt_ alone, EspD_wt_-^35^His alone or in combination with EspB_wt_, EspB_Tir1_ or EspB_Tir2_ were subjected to co-purification using Ni-beads. Samples of supernatants and elution fractions were loaded on SDS-PAGE and analyzed by western blotting with anti-His and anti-EspB antibodies. Supernatants confirmed proper protein secretion to the extracellular medium. EspB_wt_, EspB_Tir1_ or EspB_Tir2_ co-eluted with EspD_wt_-^35^His

**EspB TMD involvement in EspB-EspD interaction**. As the interaction between major and the minor translocators has been reported to occur prior to membrane integration [8,9,25,66], we examined whether the impaired translocation we observed for EspB_Tir1_ and EspB_Tir2_ resulted from defective EspB-EspD interaction. For that purpose, we grew EPEC Δ*espB* that express EspD_wt_-^35^His alone or in the presence of EspB_wt_ (EspD and EspB were expressed using two different plasmids) under T3SS-inducing conditions. EPEC Δ*espB* that expressed only EspB_wt_ was used as a negative control. The supernatants, containing T3SS secreted proteins, were collected and incubated with Ni-NTA resin to pull down EspD_wt_-^35^His along with its binding partners. SDS-PAGE and western blot analysis of bacterial supernatants and eluted samples, using anti-His and anti-EspB antibodies, demonstrated that EspB co-eluted with EspD_wt_-^35^His prior to their host membrane integration (Figure 7), as previously reported [8,25]. Using the same experimental setup, we examined if replacement of EspB TMD by each of the Tir TMDs disrupts the EspB-EspD interaction. We showed that both EspB_Tir1_ and EspB_Tir2_ co-eluted with EspD_wt_-^35^His (Figure 7), suggesting that the EspB TMD sequence is not critical to the EspB-EspD interaction that occurs prior to membrane integration.

## Discussion

TMD-containing secreted proteins bear an intrinsic conflict as, on the one hand, they include a signal for extracellular secretion while, on the other hand they contain TMDs that target them for integration into the bacterial membrane. It was recently shown that type III secreted TMD-containing proteins contain only one or two TMDs and that these TMDs are usually of moderate hydrophobicity [46]. The moderate hydrophobicity was suggested to prevent erroneous integration of the proteins into the bacterial membrane even when expressed in mutant strains that were deficient in their T3SS activity. Moreover, incorporation of isolated TMDs into two reporter systems revealed that these TMDs could not facilitate membrane integration to a similar extent as TMDs of classical transmembrane proteins. This finding suggested that the TMD sequences of secreted proteins have a crucial role in preventing the integration of these proteins into the bacterial membrane [46]. In this study, we utilized two TMD-containing secreted proteins, EspB and EspD, to examine the role of their TMDs in the ability of the proteins to be secreted by the T3SS. In contrast to the previous study, we followed the secretion and localization of the native proteins and compared them to their TMD-exchanged versions. Based on the Wimley-White water/octanol free energy scale [54,55], which predicts that segments composed of leucine and alanine residues will be stably inserted across the membrane [67], we replaced the 16 central residues of each TMD by a 7L9A sequence. Our results demonstrated that replacement of TMDs of EspB or EspD by the 7L9A sequence results in severely attenuated protein secretion and inaccurate localization of the full-size EspB and EspD proteins in the bacterial membrane (Figure 1-3). As the replacement of the native TMD core sequences by the 7L9A sequence increases the local hydrophobicity [68,69], our results support the previously suggested hypothesis that the moderate hydrophobicity of TMDs of secreted proteins facilitates their secretion [46]. Interestingly, we observed that expression of EspB_7L9A_-His in the WT EPEC strain partially inhibited native EspB secretion (Figure 1c). This dominant-negative effect might suggest that EspB_7L9A_-His competes with the native EspB for chaperone binding, and therefore slows down EspB secretion. This conclusion was further supported by our finding that the EspB_7L9A_ interacted with the CesAB chaperone to a similar level as the EspB_wt_ form (Figure 2c).

To determine if the TMDs of TMD-containing secreted proteins have common features that support their secretion, we asked if the TMD of EspB can be replaced by that of Tir, an additional type III TMD-containing secreted protein. Since Tir has two TMDs, while EspB has only one, we created two EspB replacement variants, one with the first TMD of Tir (Tir1) and the other with the second TMD of Tir (Tir2). In contrast to EspB_7L9A_-His protein, both EspB_Tir1_-His and EspB_Tir2_-His were secreted to a similar extent as EspB_wt_-His when expressed within the Δ*espB* (Figure 4). These results suggest that the TMDs of secreted proteins are not classical TMDs and that they contain unique features (e.g., hydrophobicity level, length, specific amino acid motifs, chaperone binding site). These features enable them to escape recognition by the SRP machinery that identifies membrane proteins during their translation and delivers them to the membrane, where co-translational translocation occurs [37–39].

Upon secretion of TMD-containing proteins to the extracellular medium, they are required to interact with their binding partners, adopt transmembrane orientation, and perform their function within the host membrane. To determine if the TMDs are involved in these processes, we examined whether EspB_Tir1_-His and EspB_Tir2_-His, which display similar secretion levels as EspB_wt_-His, complement the translocation activity of the Δ*espB* mutant strain. While the Δ*espB* EPEC strain can secrete the EspA and EspD translocators, it is deficient in its ability to translocate effectors into the host cells and cause disease [16,70,71]. Complementation of the Δ*espB* phenotype was only achieved when EspB_wt_ was unlabeled (Fig. S1). Therefore, we cloned unlabeled EspB with Tir1 or Tir2 variants and examined their ability to complement NleD translocation. By following the degradation pattern of the NleD substrate, JNK, we observed that neither EspB_Tir1_ nor EspB_Tir2_ were able to complement the Δ*espB* phenotype (Figure 5), suggesting that the TMD of EspB is critical for EspB function post-secretion and cannot be replaced by an alternative moderate hydrophobic sequence.

Examination of the involvement of EspB TMD in the ability of the protein to translocate into host cells was experimentally challenging as EspB was previously reported to translocate in low amounts into the host membrane and was visible only after long exposure [72]. Using a strong rat anti-EspB antibody, we detected EspB translocation into host cells by WT EPEC and Δ*espB* strain that expresses EspB_wt_ protein (Figure 6). On the other hand, cells infected with Δ*espB* that express either EspB_7L9A_, EspB_Tir1_ or EspB_Tir2_ showed no EspB translocation (Figure 6), thus suggesting that EspB TMD is critical for correct integration of the full-length protein into the host cell membrane. Unexpectedly, we observed severe reduction of EspD translocation into host cell membrane of cells infected with Δ*espB* that express either EspB_7L9A_, EspB_Tir1_ or EspB_Tir2_, although considerate EspD levels were found in cells infected by the Δ*espB* strain mutant (Figure 6). These results might suggest that the presence of mutant EspB protein that remains in the extracellular fraction prevents translocation of EspD into host cells, probably due to EspB-EspD interaction prior to their membrane integration (Figure 7). Host cells that showed no EspB and EspD translocation, also lacked the sequential Tir translocation (Figure 6).

To examine whether the impaired translocation of TMD-exchanged EspB versions resulted from defective EspB-EspD interaction, which was reported to occur prior to membrane association, we examined whether replacement of EspB TMD disrupted EspB-EspD interactions. We found that both EspB_Tir1_ and EspB_Tir2_ preserve the protein interaction with EspD_wt_-^35^His (Figure 7), thus excluding the possibility that these exchanged proteins failed to complement the Δ*espB* strain due to their inability to interact with EspD. This result correlates well with a previous study that demonstrated that the EspB-EspD interaction is mediated by regions found at either side of EspB TMD but not within the TMD itself [8].

We attempted to determine whether the impaired EspB translocation of TMD-exchanged variants was due to defective self-oligomerization. Unfortunately, we could not detect formation of EspB_wt_-His self-oligomers under soluble or membrane-simulating conditions (data not shown) and therefore we could not assess the involvement of EspB TMD in the ability of the protein to self-oligomerize. This finding might be related to the presence of an His-tag at the protein C-terminal, which was shown to prevent the proper complementation of Δ*espB* infection and translocation activity of the strain (Fig. S1), or the requirement for the presence of the EspD protein. Further investigation is required to characterize the conditions that stimulate EspB self/hetero-oligomerization before we can determine if the TMD of EspB is involved in this process.

Overall, our study suggests that the TMD sequences of TMD-containing secreted proteins encode information that differentiates them from classical TMDs and allows their cognate proteins to be secreted rather than integrated into the bacterial membrane. Moreover, TMDs of TMD-containing secreted proteins are not necessarily interchangeable, as they are involved in the ability of the proteins to associate and translocate into the host membrane and support translocation of T3SS effectors.

## Material and methods:

**Bacterial strains**. Wild-type EPEC O127:H6 strain E2348/69 [streptomycin-resistant] [73] and EPEC null mutants (Δ*escN*, Δ*espB* and Δ*espD*) were used to assess the T3SS and translocation activities [7,18,74]. *E.coli* DH10B was used for plasmid handling. The *E. coli* strains (Table S1) were grown at 37^°^C in Luria-Bertani (LB) broth (Sigma) supplemented with the appropriate antibiotics. Antibiotics were used at the following concentrations: streptomycin (50 μg/mL), ampicillin (100 µg/mL), and chloramphenicol (30 μg/mL).

**Construction of plasmids expressing labeled and unlabeled EspB_wt_ and TMD-exchanged EspB versions**. Cloning was done using the Gibson assembly method [75,76]. Briefly, the *espB* gene was amplified from EPEC genomic DNA using the primer pairs EspB_Gib_F/EspB_His_Gib_R (Table S2), which fused a Hexa His-tag to the coding region of *espB*. The pSA10 plasmid was amplified with the primer pair pSA10_F/pSA10_R (Table S2). The PCR products were subjected to digestion with *Dpn*I, purified, and assembled by the Gibson assembly method. Cloning of an untagged version of EspB was done by inserting a stop codon before the Hexa His-tag by performing site-directed mutagenesis using the primer pair EspB_His_mut_F/EspB_His_mut_R (Table S2). The tagged and untagged versions of *espB* were similarly cloned into the pACYC184 plasmid [77] by amplifying the labeled gene from the pSA10 vectors using the primer pairs EspB_pACYC_F/EspB_pACYC_His_R or EspB_pACYC_F/EspB_pACYC_R, respectively (Table S2) and the pACYC184 vector using the primer pair pACYC _F/pACYC _R (Table S2). PCR products were subjected to digestion with *Dpn*I, purified, and assembled by the Gibson assembly method. The resulting constructs, pEspB_wt,_ and pEspB_wt_-His in either the high copy number plasmid (pSA10) or the low copy number plasmid (pACYC184), expressed untagged EspB or EspB fused to a C-terminal His-tag.

The TMD-exchanged versions of *espB* were initially generated using the pEspB_wt_-His (pSA10) vector as a template. To replace the TMD of EspB by a TMD backbone sequence of 7-leucine-9-alanine (7L9A), the EspB 1–100 amino acid sequence was amplified using the primer pair EspB_Gib_F/EspB1_100_Gib_R (Table S2) from the pEspB_wt_-His vector. The TMD 7L9A backbone was generated by annealing the primer pair 7L9AF/7L9AR (Table S2) by heating the sample to 95°C for 5 min and then decreasing the temperature to 20°C at a rate of 5°C/min. The 7L9A backbone was then amplified using the primer pair EspB7L9AF/EspB7L9AR. The EspB_1-100_ PCR fragment and the 7L9A backbone were then ligated using overlapping sequences and amplified using the primer pair EspB_Gib_F/EspB7L9AR (Table S2). Gibson assembly was conducted by amplifying the pEspB_wt_-His pSA10 vector with the primer pair EspB_TMexF/pSA10_R (Table S2), followed by *Dpn*I treatment of the reaction and subjecting the amplified vector and the EspB_1-100_-7L9A fused PCR fragment to ligation. The resulting construct, pEspB_7L9A_-His (pSA10), expressed EspB-His protein that contains a 7L9A sequence instead of the original TMD sequence.

To replace the original TMD of EspB with either the first (Tir1) or second (Tir2) TMDs of Tir, the TMD were amplified from EPEC genomic DNA using the primer pairs EspBTirTMDex1_F/EspBTirTMDex1_R and EspBTirTMDex2_F/EspBTirTMDex2_R, respectively (Table S2). In parallel, the EspB fragment following EspB TMD (amino acids 120–220 of the EspB sequence) was amplified using the primer pair EspB100aaTMD_F/EspB100aaTMD_R (Table S2) and then ligated to Tir1 or Tir2 using overlapping sequences and amplified using the primer pairs EspBTirTMDex1_F/EspB100aaTMD_R or EspBTirTMDex2_F/EspB100aaTMD_R, respectively (Table S2). Gibson assembly was conducted by amplifying the pEspB_wt_-His pSA10 vector with the primer pair EspB_TMD_open_F/EspB_TMD_open_R (Table S2), followed by *Dpn*I treatment of the reaction and subjecting the amplified vector and either Tir1-EspB_120-220_ or Tir2-EspB_120-220_ fused PCR fragments to ligation. The resulting constructs, pEspB_Tir1_-His (pSA10) and pEspB_Tir2_-His (pSA10), expressed EspB-His protein that contains either the first or the second TMD sequence of Tir instead of the original EspB TMD sequence. Cloning of an untagged versions of EspB_Tir1_ and EspB_Tir2_ was done by inserting a stop codon before the Hexa His-tag by performing site-directed mutagenesis using the primer pair EspB_His_mut_F/EspB_His_mut_R (Table S2). To clone the untagged versions of EspB_Tir1_ and EspB_Tir2_ into a low copy plasmid pACYC184, Gibson assembly was conducted by amplifying the pEspB_wt_ pACYC184 vector with the primer pair EspB_TMD_open_F/EspB_TMD_open_R (Table S2), followed by *Dpn*I treatment of the reaction and subjecting the amplified vector and either Tir1-EspB_120-220_ or Tir2-EspB_120-220_ fused PCR fragments to ligation. The resulting constructs, pEspB_Tir1_ (pACYC184) and pEspB_Tir2_ (pACYC), expressed an untagged EspB protein that contains either the first or the second TMD sequence of Tir instead of the original EspB TMD sequence. All constructs were verified by DNA sequencing.

**Construction of plasmids expressing labeled EspD_wt_ and TMD-exchanged EspD versions**. The EspD protein was tagged with a Hexa His at position 35, to avoid disruption of its function. The fragment encoding amino acids 1–35 of EspD was amplified from EPEC genomic DNA by using the primer pairs EspD_Gib_F/EspD35_His_ R (Table S2), which fused a Hexa His- tag at position 35 of the EspD protein. In parallel, the fragment encoding the EspD protein starting from position 36 was amplified from EPEC genomic DNA using the primer pairs EspD35_His_ F/EspD_Gib_R (Table S2). The two fragments were ligated using overlapping sequences and amplified using the primer pair EspD_Gib_F/EspD_Gib_R (Table S2). The pSA10 plasmid was amplified with the primer pair pSA10_F/pSA10_R (Table S2). The PCR products were subjected to digestion with *Dpn*I, purified, and assembled by the Gibson assembly method. The resulting plasmid, pEspD_wt_-^35^His (pSA10), expressed EspD labeled with an internal His-tag at position 35.

The TMD-exchanged *espD* versions in pSA10 were generated with the template of pEspD_wt_-^35^His (pSA10). To replace the TMDs of EspD (TMD1 or TMD2) with the 7L9A sequence, the TMD 7L9A backbone was amplified using the primer pair EspD7L9A1F/EspD7L9A1R for exchanging the first TMD of EspD and the primer pair EspD7L9A2F/EspD7L9A2R for exchanging the second TMD of EspD (Table S2). In parallel, the EspD coding region upstream of the EspD TMD1 (amino acids 1–184 of the EspD sequence) was amplified using the primer pair EspD_Gib_F/EspD184aa_R (Table S2) and the EspD coding region downstream of the EspD TMD2 (amino acids 253–380 of the EspD sequence) was amplified using the primer pair EspD253aa_F/EspD_Gib_R (Table S2). For EspD TMD1 exchange, the fragment encoding EspD1-184 was then ligated to the 7L9A TMD1 backbone using overlapping sequences, and amplified with the primer pair EspD_Gib_F/EspD7L9A1R (Table S2). Gibson assembly was conducted by amplifying the pEspD_wt_-^35^His pSA10 vector with the primer pair EspD_TMD1_open_F/pSA10_R (Table S2), followed by *Dpn*I treatment of the reaction and subjecting the amplified vector and EspD_1-184_-TMD1 to ligation. For the EspD TMD2 exchange, the 7L9A TMD2 backbone was ligated to the fragment encoding the EspD253-380 sequence using overlapping sequences and amplified with the primer pair EspD7L9A2F/EspD_Gib_R (Table S2). Gibson assembly was conducted by amplifying the pEspD_wt_-^35^His pSA10 vector with the primer pair pSA10_F/EspD_TMD2_open_R (Table S2), followed by *Dpn*I treatment of the reaction and subjecting the amplified vector and TMD2-EspD_253-380_ to ligation. The resulting plasmid, pEspD_wt_-^35^His (pSA10) and pEspD_wt_-^35^His (pSA10), expressed EspD labeled with an internal His-tag at position 35 that contains a 7L9A sequence instead of TMD1 or TMD2 of the original EspD TMDs, respectively. All constructs were verified by DNA sequencing.

**Construction of a plasmid expressing CesAB-His**. The fragment encoding *cesAB* gene was amplified from EPEC genomic DNA by using the primers CesAB_F/CesABHis_R1 and then CesAB_F/CesABHis_R2 (Table S2), which fused a C-terminal hexa-His tag to the open reading frame of the protein. The pSA10 plasmid was amplified using the primer pair pSA10_F/pSA10_R (Table S2). The PCR products were subjected to digestion with *Dpn*I, purified, and assembled by the Gibson assembly method.

***In vitro* type III secretion assay**. EPEC strains were grown overnight in LB supplemented with the appropriate antibiotics, in a shaker at 37^°^C. The cultures were diluted 1:40 into pre-heated Dulbecco’s modified Eagle’s medium (DMEM, Biological Industries) supplemented with the appropriate antibiotics, and grown statically for 6 h in a tissue culture incubator (with 5% CO_2_), to an optical density of 0.7 at 600 nm (OD_600_). To induce protein expression, 0.5 mM IPTG was added to bacterial cultures. The cultures were then centrifuged at 20,000 × *g* for 5 min to separate the bacterial pellets from the supernatants; the pellets were dissolved in SDS-PAGE sample buffer, and the supernatants were collected and filtered through a 0.22 μm filter (Millipore). The supernatants were then precipitated with 10% (v/v) trichloroacetic acid (TCA) overnight at 4^°^C to concentrate proteins secreted into the culture medium. The volume of the supernatants was normalized to the bacterial cultures at OD_600_ to ensure equal loading of the samples. The samples were then centrifuged at 18,000 × *g* for 30 min at 4^°^C, the precipitates of the secreted proteins were dissolved in SDS-PAGE sample buffer, and the residual TCA was neutralized with saturated Tris. Proteins were analyzed on 12% SDS-PAGE gels and stained with Coomassie blue.

**Immunoblotting**. Samples were subjected to SDS-PAGE and transferred to nitrocellulose (pore size: 0.45 µm; Bio-Rad) or PVDF (Mercury, Millipore) membranes. The blots were blocked for 1 h with 5% (w/v) skim milk-PBST (0.1% Tween in phosphate-buffered saline), incubated with the primary antibody (diluted in 5% skim milk-PBST for 1 h at room temperature or overnight at 4^°^C), washed and then incubated with the secondary antibody (diluted in 5% skim milk-PBST, for 1 h at room temperature). Chemiluminescence was detected with EZ-ECL reagents (Biological Industries). The following primary antibodies were used: mouse anti-His (Pierce), diluted 1:2000; rabbit anti-MBP (ThermoFisher Scientific), diluted 1:1000; mouse anti-JNK (BD Pharmingen), diluted 1:1000 in TBST; mouse anti-DnaK (Abcam, Inc.), diluted 1:5000; and mouse anti-actin (MPBio), diluted 1:10,000. Antibodies directed against T3SS components were a generous gift from Prof. B. Brett Finlay (University of British Columbia, Canada) and Prof. Rebekeh DeVinney (University of Calgary, Canada) and included mouse anti-EspB, mouse anti-Tir, rat anti-intimin, rat anti-EspB, and rat anti-EspD. The following secondary antibodies were used: horseradish peroxidase-conjugated (HRP)-goat anti-mouse (Abcam Inc.), HRP-conjugated goat anti-rabbit (Abcam Inc.), and HRP-conjugated goat anti-rat (Jackson ImmunoResearch) antibodies.

**Bacterial fractionation**. Bacterial cell fractionation was performed based on a previously described procedure [74]. Briefly, EPEC strains from an overnight culture were sub-cultured 1:50 in 50 mL DMEM, and grown statically for 6 h, at 37^°^C, in a CO_2_ tissue culture incubator. Cells were harvested, washed in PBS, and resuspended in 0.25 mL buffer A [50 mM Tris (pH 7.5), 20% (w/v) sucrose, 5 mM EDTA, protease inhibitor cocktail (Roche Applied Science), and lysozyme (100 µg/mL)] and incubated with rotation for 15 min at room temperature to generate spheroplasts. MgCl_2_ was then added to a final concentration of 20 mM, and samples were centrifuged for 10 min at 5000 × *g*. The supernatants containing the periplasmic fractions were collected. The pellets, which contained the cytoplasm and the membrane fractions, were resuspended in 1 mL lysis buffer (20 mM Tris/HCl pH 7.5, 150 mM NaCl, 3 mM MgCl_2_, 1 mM CaCl_2_, and 2 mM β-mercaptoethanol with protease inhibitors). All subsequent steps were carried out at 4^°^C. RNase A and DNase I (10 µg/mL) were added, and the samples were sonicated (Fisher Scientific, 3 × 15 s). Intact bacteria were removed by centrifugation (2300 × *g* for 15 min), and the cleared supernatants containing cytoplasmic, and membrane proteins were transferred to new tubes. To obtain the cytoplasmic fraction, the supernatants were centrifuged (in a Beckman Optima XE-90 Ultracentrifuge with an SW60 Ti rotor) for 30 min at 100,000 × *g*, to pellet the membranes. The supernatants, containing the cytoplasmic fraction, were collected and the pellets, containing the membrane fractions, were washed with lysis buffer and resuspended in 0.1 mL lysis buffer with 0.1% SDS. The protein content of all samples was determined using the Coomassie Plus protein assay (Thermo Scientific) before adding SDS-PAGE sample buffer with β-mercaptoethanol. Intimin, maltose-binding protein (MBP), and DnaK were used as markers for the membrane, periplasm and cytoplasm fractions, respectively.

**Translocation activity**. Translocation assays were performed as previously described [64]. Briefly, HeLa cells (Porgador laboratory, BGU, 8 × 10^5^ cells per well) were infected for 3 h with EPEC strains that were pre-induced for 3 h for T3SS activity (pre-heated DMEM, statically, in a CO_2_ tissue culture incubator). Cells were then washed with PBS, collected, and lysed with RIPA buffer. Samples were centrifuged at maximum speed for 5 min to remove non-lysed cells, and supernatants were collected, mixed with SDS-PAGE sample buffer, and subjected to western blot analysis with anti-JNK and anti-actin antibodies (loading control). Uninfected samples and the Δ*escN* mutant strain-infected samples were used as negative controls. EspB, EspD and Tir translocation was performed as previously described [8]. Briefly, HeLa cells (4 x 10^6^ in 10-cm-diameter dishes) were infected for 2 h with EPEC strains that were pre-induced for 3 h for T3SS activity at MOI 200:1. Infected cells were washed three times with cold PBS, scraped, and resuspended in 5 mL cold PBS. Cells were collected by centrifugation (1400 × *g* for 4 min at 4^°^C) and lysed in Triton X-100 buffer (50 mM Tris-HCl/pH 7.5, 1% Triton X-100 and protease inhibitors). Sample were centrifugated (16,000 × *g* for 30 min at 4^°^C) and supernatants were collected, boiled with SDS-PAGE sample buffer and subjected to western blot analysis with anti-EspB, anti-EspD, anti-Tir and anti-actin antibodies.

**Co-elution by nickel affinity chromatography**. Co-elution assays were performed as previously described [8,78]. For EspB-EspD interaction, EPEC Δ*espB* that express EspB_wt_ alone, EspD-^35^His alone or in the presence of EspB_wt_, EspB_Tir1_, or EspB_Tir2_ were grown under T3SS-inducing conditions for 6 h. The supernatants, containing secreted EspD-^35^His with or without EspB, were collected by centrifugation (20,000 × *g* for 5 min) and were passed through a 0.45-μm-pore-size filter. Protein inhibitor solution was added to the samples (1 mM PMSF), and they were incubated rotating with Ni-NTA resin at 4°C overnight. The samples were then loaded on gravity columns, and the flow-through was collected. The columns were washed five times with 5 mL of wash buffer (30 mM phosphate buffer pH 7.5, 500 mM NaCl, 50 mM imidazole), and proteins were eluted using elution buffer (30 mM phosphate buffer pH 7.5, 500 mM NaCl, 500 mM imidazole). Equal volumes of the supernatant and the eluate samples were precipitated with 10% (v/v) TCA for 1 h at 4°C, centrifuged (30 min, 16,000 × *g*, 4°C), air dried, dissolved in SDS-PAGE sample buffer and the residual TCA was neutralized with saturated Tris. To examine the EspB-CesAB interaction we used similar assay with the following modifications: EPEC Δ*espB* that express EspB_wt_ alone, CesAB-His alone or in the presence of EspB_wt_ or EspB_7L9A_ were grown in LB with 0.25 mM IPTG until reached an OD_600_ of 0.8–1.0. Growth of EPEC in LB is not inducing T3SS and therefore EspB remains within the bacteria. Bacterial cells were collected by centrifugation (20,000 × *g* for 5 min), washed in PBS, and resuspended in wash buffer with lysozyme (1 mg/mL) and incubated for 30 min in ice. The samples were than sonicated (Fisher Scientific, 3 × 10 s), intact bacteria were removed by centrifugation (15,000 × *g* for 30 min at °C), and the cleared supernatants were collected and were passed through a 0.45 μm-pore-size filter. Protease inhibitor cocktail was added to the filtrate, the samples and were incubated rotating with Ni-NTA resin at 4°C overnight. All subsequent steps were carried out similarly to the EspB-EspD interaction assay.

## Supplementary Material

Supplemental Material
