# Supplementary material for: Transmembrane domains of type III-secreted proteins affect bacterial-host interactions in enteropathogenic E. coli
Source: Virulence. 2021 Mar 17;12(1):902–17. doi: 10.1080/21505594.2021.1898777 (PMC7993127; doi:10.1080/21505594.2021.1898777)
Supplement: Supplemental Material [file KVIR_A_1898777_SM6856.docx]

**Supplemental Material**


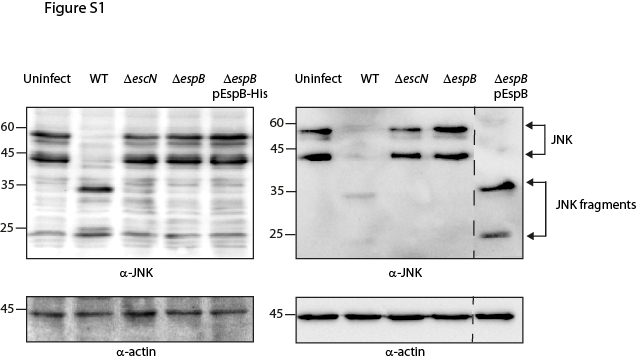


Figure S1: **Unlabeled EspB protein can complement ΔespB translocation.** Proteins extracted from HeLa cells infected with WT, Δ*escN,* Δ*espB,* and Δ*espB* expressing either EspB_wt_-His or unlabeled EspB_wt_. The samples were subjected to western blot analysis using anti-JNK and anti-actin (loading control) antibodies. JNK and its degradation fragments are indicated at the right of the gel. WT EPEC showed massive degradation of JNK relative to the uninfected sample and the samples infected with Δ*escN* or Δ*espB* mutant strains. Only the EPEC Δ*espB* strain complemented with unlabeled EspB_wt_ a showed a similar JNK degradation profile as WT EPEC.

**Table S1**: Strains and plasmids used in this study

| **Strains** | **Description** | **Reference** |
| --- | --- | --- |
| Wild-type EPEC | EPEC strain E2348/69, streptomycin resistant | (1) |
| EPEC ΔescN | Non-polar deletion of escN | (2) |
| EPEC ΔespB | Non-polar deletion of espB | (3) |
| EPEC ΔespD | Non-polar deletion of espD | (4) |
| *E. coli* DH10B | For plasmid handling | (5) |
| **Plasmids** |  |  |
| pEspB_wt_-His (pSA10) | C-terminal His tagged EspB in pSA10 | This study |
| pEspB_wt_-His (pACYC184) | C-terminal His tagged EspB in pACYC184 | This study |
| pEspB_wt_ (pSA10) | Untagged EspB in pSA10 | This study |
| pEspB_wt_ (pACYC184) | Untagged EspB in pACYC184 | This study |
| pEspB_7L9A_-His (pSA10) | C-terminal tagged EspB with a 7L9A sequence instead of the original core TMD in pSA10 | This study |
| pEspD_wt_-^35^His (pSA10) | His tagged EspD (at position 35) in pSA10 | This study |
| pEspD_TMD1_-^35^His (pSA10) | His tagged EspD (at position 35) with an 7L9A sequence instead of the original TMD1 in pSA10 | This study |
| pEspD_TMD2_-^35^His (pSA10) | His tagged EspD (at position 35) with an 7L9A sequence instead of the original TMD2 in pSA10 | This study |
| pEspB_Tir1_-His (pSA10) | C-terminal tagged EspB with the sequence of TMD1 of Tir instead of the original EspB TMD in pSA10 | This study |
| pEspB_Tir2_-His (pSA10) | C-terminal tagged EspB with the sequence of TMD2 of Tir instead of the original EspB TMD in pSA10 | This study |
| pEspB_Tir1_ (pACYC184) | Untagged EspB with the sequence of TMD1 of Tir instead of the original EspB TMD in pACYC184 | This study |
| pEspB_Tir2_ (pACYC184) | Untagged EspB with the sequence of TMD1 of Tir instead of the original EspB TMD in pACYC184 | This study |
| pEspB_Tir1_ (pSA10) | EspB with the sequence of TMD1 of Tir instead of the original EspB TMD in pSA10 | This study |
| pEspB_Tir2_ (pSA10) | EspB with the sequence of TMD2 of Tir instead of the original EspB TMD in pSA10 | This study |

**Table S2:** Sequences of primers designed and used in this study

| **Constructs and primers** | **Primer sequence** |
| --- | --- |
| **pEspB_wt_-His (pAS10)** |  |
| EspB_Gib_F | CACACAGGAAACAGatgaatactatcgataataacaatgcgg |
| EspB_His_Gib_R | GGATCCCCGGGAATTTCAGTGGTGGTGGTGGTGGTGcccagctaagcgagcc |
| pSA10_F | AATTCCCGGGGATCCGTCG |
| pSA10_R | CTGTTTCCTGTGTGAAATTGTTATCCG |
| **pEspB_wt_ (pAS10)** |  |
| EspB_His_mut_F | gcggctcgcttagctgggTAGCACCACCACCACCACTG |
| EspB_His_mut_R | CAGTGGTGGTGGTGGTGCTAcccagctaagcgagccgc |
| **pEspB_wt_-His (pACYC184)** |  |
| EspB_pACYC_F | CACCAGGATGAATAAAATTTAAAAatgaatactatcgataataacaatgcg |
| EspB_pACYC_His_R | CTCAAGGGCATCGGTCGACtcagtggtggtggtggtggtgcccagctaagcgagcc |
| pACYC_F | GTCGACCGATGCCCTTG |
| pACYC_R | ttttaaattttattcatcctggtggttg |
| **pEspB_wt_ (pACYC184)** |  |
| EspB_pACYC _R | CTCAAGGGCATCGGTCGACctacccagctaagcgagc |
| **pEspB_7L9A_-His (pSA10)** |  |
| EspB1_100_Gib_R | GTAAGAGTAGCAACAGagcggctgtcgcaccgg |
| 7L9AF | CTGTTGCTACTCTTACTCCTTGCGGCCGCAGCGGCTGCAGCGGCAGCC |
| 7L9AR | GGCTGCCGCTGCAGCCGCTGCGGCCGCAAGGAGTAAGAGTAGCAACAG |
| EspB7L9AF | gccggtgcgacagccgctCTGTTGCTACTCTTACTCCttgcgg |
| EspB7L9AR | ctttagttgcgctattaatggcagcGGCTGCCGCTGCagc |
| EspB_TMexF | gctgccattaatagcgcaac |
| **pEspB_Tir1_-His (pSA10)** |  |
| EspBTirTMDex1_F | gatgagaaaaaagccggtTTCTGGGTTTCTGTCGGCG |
| EspBTirTMDex1_R | cgcgcctttagttgcgctattAATACCAGTTGCCGCCAG |
| EspB100aaTMD_F | aatagcgcaactaaaggcgcgagtgatgtcgc |
| EspB100aaTMD_R | ggcagcagcagtaaagcgacttaataatcctgc |
| EspB_TMD_open_F | agtcgctttactgctgctgccgg |
| EspB_TMD_open_R | accggcttttttctcatcaatagc |
| **pEspB_Tir2_-His (pSA10)** |  |
| EspBTirTMDex2_F | gatgagaaaaaagccggtATTGGTTACGGCCTCAGC |
| EspBTirTMDex2_R | cgcgcctttagttgcgctattGAGCGCAGTCGTTACACC |
| **pEspD_wt_-^35^His** |  |
| EspD_Gib_F | CACACAGGAAACAGatgcttaatgtaaataacgatatcc |
| EspD35_His_R | GTGGTGGTGGTGGTGGTGtaaatccagcgcggaagtc |
| EspD35_His_F | CACCACCACCACCACCACcaactggttaaatccacgg |
| EspD_Gib_R | CGGATCCCCGGGAATTttaaactcgaccgctgac |
| **pEspD_TMD1_-^35^His (pSA10)** |  |
| EspD7L9A1F | ggtcaggtctttggttggCTGTTGCTACTCTTACTCCTTGCGG |
| EspD7L9A1R | actaatagcaacaacagcccaGGCTGCCGCTGCAGC |
| EspD184aa_R | GGAGTAAGAGTAGCAACAGccaaccaaagacctgaccaac |
| EspD_TMD1_open_F | tgggctgttgttgctattagt |
| **pEspD_TMD2_-^35^His (pSA10)** |  |
| EspD7L9A2F | gcaagcttttggcggtCTGTTGCTACTCTTACTCCTTGCGG |
| EspD7L9A2R | gacatcgccaactttagatataagtgaagaGGCTGCCGCTGCAGC |
| EspD253aa_F | GCTGCAGCGGCAGCCtcttcacttatatctaaagttggcgatgtc |
| EspD_TMD2_open_R | accgccaaaagcttgc |

**References**

1. Iguchi A, Thomson NR, Ogura Y, Saunders D, Ooka T, Henderson IR, Harris D, Asadulghani M, Kurokawa K, Dean P, Kenny B, Quail MA, Thurston S, Dougan G, Hayashi T, Parkhill J, Frankel G. 2009. Complete genome sequence and comparative genome analysis of enteropathogenic *Escherichia coli* O127:H6 strain E2348/69. J Bacteriol 191:347-354.

2. Gauthier A, Puente JL, Finlay BB. 2003. Secretin of the enteropathogenic *Escherichia coli* type III secretion system requires components of the type III apparatus for assembly and localization. Infect Immun 71:3310-9.

3. Luo W, Donnenberg MS. 2006. Analysis of the function of enteropathogenic *Escherichia coli* EspB by random mutagenesis. Infect Immun 74:810-820.

4. Tseytin I, Dagan A, Oren S, Sal-Man N. 2018. The role of EscD in supporting EscC polymerization in the type III secretion system of enteropathogenic *Escherichia coli*. Biochim Biophys Acta Biomembr 1860:384-395.

5. Durfee T, Nelson R, Baldwin S, Plunkett G, 3rd, Burland V, Mau B, Petrosino JF, Qin X, Muzny DM, Ayele M, Gibbs RA, Csorgo B, Posfai G, Weinstock GM, Blattner FR. 2008. The complete genome sequence of *Escherichia coli* DH10B: insights into the biology of a laboratory workhorse. J Bacteriol 190:2597-2606.
